# Molecular Characterization and Viral Origin of the 2015 Dengue Outbreak in Xishuangbanna, Yunnan, China

**DOI:** 10.1038/srep34444

**Published:** 2016-09-29

**Authors:** Yujiao Zhao, Lihua Li, Dehong Ma, Jia Luo, Zhiqiang Ma, Xiaodan Wang, Yue Pan, Junying Chen, Juemin Xi, Jiajia Yang, Lijuan Qiu, Chunhai Bai, Liming Jiang, Xiyun Shan, Qiangming Sun

**Affiliations:** 1 Institute of Medical Biology, Chinese Academy of Medical Sciences, and Peking Union Medical College, Kunming 650118, PR China; 2 Yunnan Key Laboratory of Vaccine Research & Development on Severe Infectious Diseases, Kunming 650118, PR China; 3 Xishuangbanna Dai Autonomous Prefecture People’s Hospital, Jinghong 666100, PR China; 4 Kunming Medical University, Kunming 650500, PR China

## Abstract

A total of 1067 serum samples were collected from febrile patients in Xishuangbanna, Yunnan, 2015. Of these, 852 cases were confirmed to be dengue NS1-positive. 76 structural protein genes were sequenced through RT-PCR based on the viral RNAs extracted from serum samples. Phylogenetic analysis revealed that all strains were classified as cosmopolitan genotype of DENV-2. After comparing with the DENV-2SS, 173 base substitutions were found in 76 sequences, resulting in 43 nonsynonymous mutations, of which 22 mutations existed among all samples. According to secondary structure prediction, 8 new possible nucelotide/protein binding sites were found and another 4 sites were lost among the 775 amino acids of DENV structural proteins as compared with DENV-2SS. Meanwhile, 6 distinct amino acid changes were found in the helix and strand regions, and the distribution of the exposed and buried regions was slightly altered. The results indicated that the epidemic dengue strains of Xishuangbanna in 2015 are most similar to the Indian strain in 2001 and the Sri Lankan strain in 2004. Moreover, it also show a very strong similarity to the epidemic strains of Fujian province in 1999 and 2010, which show that there is an internal recycling epidemic trend of DENV in China.

Dengue virus (DENV) belongs to the Flavivirus genus and is transmitted by mosquitoes, such as *Aedes albopictus* and *Ae. aegypti*. There are four serotypes (DENV-1, 2, 3 and 4) that are circulating in tropical and subtropical regions and are found in over one hundred countries, threatening 2.5 billion people worldwide^1^,^2^. The WHO declared that climate change has provided DENV with a vastly expanded geographical range and that the distribution of DENV has grown dramatically worldwide over the years^3^.

Yunnan province is located in southwestern China and borders the countries of Southeast Asia where dengue fever is commonplace. Imported cases of DENV infection sporadically occur in bordering regions, such as Xishuangbanna and Dehong. The first outbreak of dengue fever (DF) in Yunnan province was reported in 2008, with 56 confirmed cases^4^. Since then, epidemics have been regularly reported in Yunnan. For example, a large scale outbreak occurred in 2013, with 1538 infected individuals, indicating the severity of the incidence of dengue infection in Yunnan^5^,^6^,^7^,^8^.

The first imported case of DF in Xishuangbanna in 2015 was reported on July 13. Subsequently, the first local infection case was reported on August 20^th^. From then until September 24th, there were 103 reported cases of DF, including 10 imported cases and 93 local cases^9^. The epidemic continued until the middle of November, at which point more than one thousand cases were confirmed. The scale of the DENV epidemic at this location is relatively rare. Thus far, detailed genetic and molecular epidemiologic information regarding the DENV strain involved in this outbreak remains elusive.

The list of countries affected by DENV outbreaks in 2015 is extensive and includes Vietnam, Myanmar, Laos, Taiwan and Guangdong of China, as well as many areas that are geographically near Yunnan. Epidemiological investigation and virus evolutionary analysis are useful tools for identifying the transmission relationships between common DENV strains in Xishuangbanna, Yunnan, other regions in China and neighboring Southeast Asian countries.

## Results

### The geographic analysis of Xishuangbanna and study design

**Figure 1:**
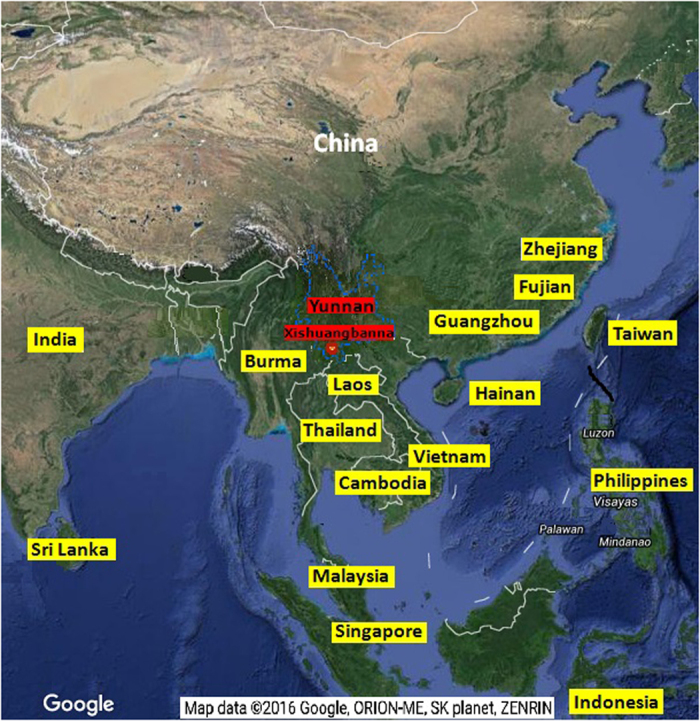
Geographic relationships between Xishuangbanna and other Asian dengue outbreak countries and areas in 2015. The screenshot of base satellite map of Asia was obtained from Google Earth (http://google.com/). The boundary of Yunnan province was marked in blue dotted line. The red oval was used to mark Xishuangbanna state of Yunnan province and the yellow block represents the other dengue epidemic areas in China and around Asia. It’s obvious that Yunnan province is geographically a central and connecting area among them, which led to a serious trend of dengue epidemic in Yunnan in recent years. (Scientific Reports remains neutral with regard to jurisdictional claims in published maps).

**Figure 2:**
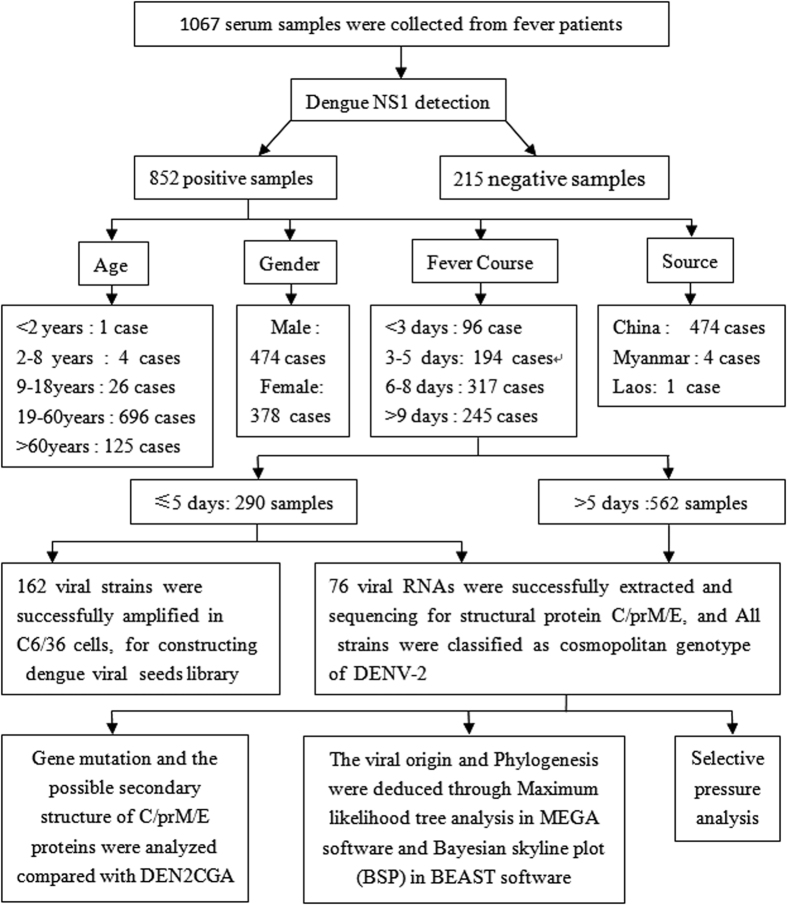
The study design and the following disposition of study subjects. 1067 patients who went to the hospital for fever were recruited in our study; among them, 852 cases were identified as dengue NS1 positive. Of these, serum samples were collected for virus amplification and viral RNAs extraction. Phylogenetic analysis was then conducted to characterize the origin and prevalence of DENV in Xishuangbanna.

The geographic relationships between Yunnan and the 2015 DENV outbreak countries and areas in Asia were analyzed first. The results show that Yunnan has become a central DENV epidemic area, as the distances between Yunnan and the other DF outbreak areas are short (Fig. 1). During the DENV outbreak in Xishuangbanna, Yunnan from August to November 2015, a total of 1067 serum samples from febrile patients were collected at the Xishuangbanna Dai Autonomous Prefecture People’s Hospital, and 852 cases were confirmed to be NS1 positive through Colloidal Gold test. Of these, 290 DENV positive serum samples were screened out from patients whose fever courses were shorter than 5 days. Of these, a total of 162 virus strains led to cytopathic effects after amplification in C6/36 cells for 7 days, in order to construct viral seeds library of Xishuangbanna DENV (XNDV). In addition, 76 viral RNAs were successfully extracted directly from these serum samples, followed by gene sequencing of the DENV structural protein C/prM/E genes. Phylogenetic analysis was then conducted through Maximum likelihood (ML) tree analysis in MEGA software and Bayesian skyline plot (BSP) in BEAST software, in order to characterize the origin and genetic relationship of the DENV in Xishuangbanna. The study design and the following disposition of study subjects are shown in Fig. 2.

### Amino acid mutations and possible secondary structure in structural protein regions

**Figure 3:**
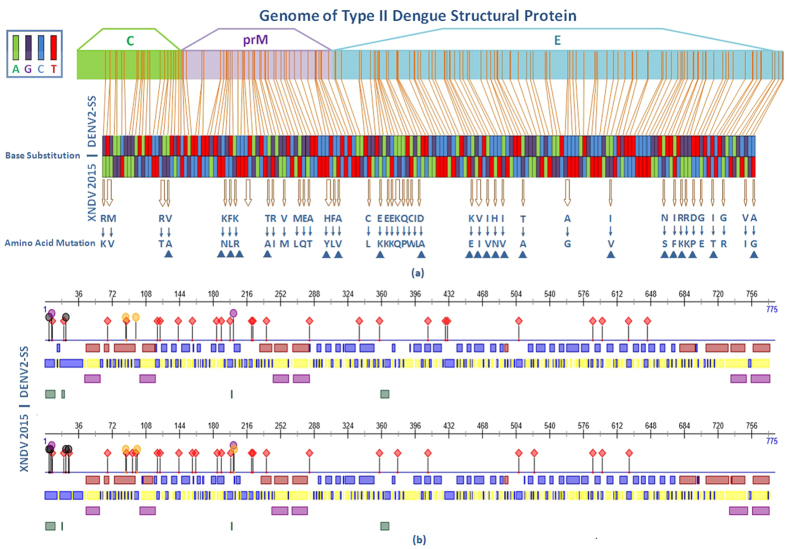
Molecular character analysis of the XNDV strains 2015 comparing with DENV-2 standard strain (Guinea 1944). (**a**) Base substitutions and amino acid mutations of the XNDV strain compared with the DEN2SS. ▲ Indicates that the mutation existed in all 76 sample strains. (**b**) Secondary structure prediction of the structural proteins for DEN2SS and XNDV. The black dot denotes nucelotide-binding region; purple dot denotes the RNA-binding region; yellow dot denotes the DNA-binding region and the red rhombus denotes the protein-binding region. Blue and red in the first line represent the strand and helix regions, respectively. Blue and yellow in the second line represent the exposed and buried regions, respectively. Purple in the third line indicates the helical transmembrane regions, and green in the fourth line represents the disordered regions.

All 76 sequences of structural protein coding regions obtained from the epidemic strains of DENV were primarily blasted against the DENV standard strains of four serotypes. All strains were identified to be most similar with serotype 2 standard strain of DENV (DENV-2SS). The analyses of the base substitutions and amino acid mutations were performed by comparison with the DEN2SS. The result shows that there were 173 base substitutions in the DENV structural protein C/prM/E genes, leading to 43 nonsynonymous mutations, of which 21 mutations appeared in only one or a few strains, whereas 22 mutations existed in all 76 sample strains. These were considered the special molecular characteristics of the epidemic-causing DENV strain in Xishuangbanna, China, 2015 (Fig. 3a). The possible secondary structure of C/prM/E proteins of XNDV 2015 were then predicted and compared with DEN2SS. There were 12 changes of possible nucelotide/protein binding sites among the 775 amino acids that form DENV structural proteins C/prM/E. Of these 8 possible protein binding sites, sites 6, 22, 93, 97, 161, 202, 376 and 522 existed only in XNDV, whereas sites 335, 427, 429 and 642 existed only in DEN2SS. Approximately 6 distinct changes were found in the helix and strand regions, and the distribution of the exposed and buried regions was slightly altered. Moreover, there was almost no difference between DEN2SS and XNDV with respect to the helical transmembrane regions and the disordered regions (Fig. 3b).

### Phylogenetic analysis

**Figure 4:**
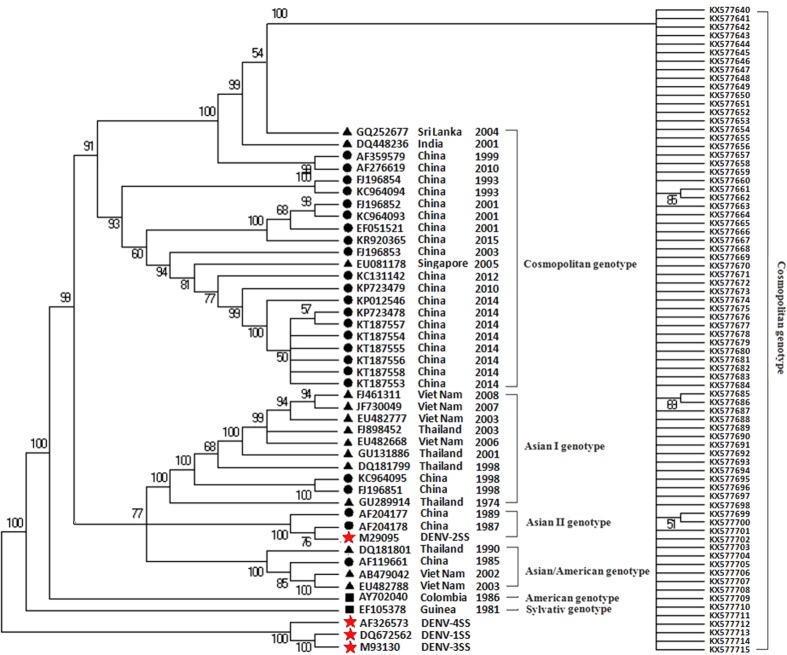
Phylogenetic tree of DENV-2 epidemic strains in Xishuangbanna, Yunnan, China 2015. The phylogenetic tree was constructed using the Maximum Likelyhood phylogeny test. Bootstrap values were set for 500 repetitions. (● denotes DENV strains from China, ▲ denotes strains from other Asian countries, and ■ denotes strains from other countries outside of Asia, and the red 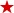 denotes the standard strains of four dengue serotypes. The sequences of the reference strains were derived from the NCBI GenBank database (www.ncbi.nlm.nih.gov).

A total of 76 XNDV envelope genes were aligned for phylogenetic analysis and compared with the reference sequences including standard strains of four serotypes, typical Chinese strains and other strains epidemic in neighboring countries derived from the GenBank database. Phylogenetically, all 76 strains of XNDV were serotype 2 DENV and were further subclassified into the cosmopolitan genotype^10^. All XNDV strains had high similarity and belong to one cluster in the ML tree. The strains from India 2001 (DQ448236) and Sri Lanka 2004 (GQ252677) were grouped in one branch and have the closest relationship to the XNDV strains. Notably, two Chinese strains (AF276619; AF359579) were located in the branch next to the XNDV cluster, of which the AF276619 strain and AF359579 strain led to the DF epidemic in Fujian province of China in 1999 and 2010 respectively (Fig. 4). Subsequently, more reference sequences were added to the phylogenetic analysis using Bayesian skyline plot (BSP) in BEAST software. The result shows high similarity to the ML tree (Supplement Fig. 1). This demonstrates that DF is becoming a locally circulating epidemic disease.

### Selective pressure analysis

The variable dN/dS rate ratios of the XNDV strains were tested comparing with 24 Asia reference lineages. There was one positively selected site in Model 2 and 2 sites in Model 8, but the P-value was >0.05, which shows no significant evidence of positive selection in the sequence alignment of the DENV E protein genes, possibly because E gene of Asian DENV strains have a conservative evolution, and the selective purification pressure on them was relatively high. The selective pressure analysis results are summarized in Table 1.

## Discussion

The trend of increasing numbers of dengue fever epidemics in China has become alarming in recent years. In particular, in 2014, the cumulative number of DENV cases in China exceeded 50,000, which outnumbered the total of DENV cases in China over the past ten years. Dengue fever is widespread in Southeast Asia, the Pacific island countries and the Caribbean, of which the Southeast Asian countries that border China (especially Yunnan province) are the most heavily affected areas, leading to an increased risk of a dengue fever importation and epidemic^11^.

Yunnan province has a population of more than 50 million people. The length of the border of Yunnan province with Myanmar, Laos and Vietnam is 4061 km. As an important bridge in the Mekong sub-regional economic cooperation alliance, Xishuangbanna of Yunnan has had close contact with Myanmar, Laos, Thailand, Vietnam and other dengue fever epidemic countries in recent years. In addition, Xishuangbanna is rich in tourism resources and attracts more than 14 million tourists from all over the world annually, which also leads to an increased risk of a DENV epidemic^12^. The timely monitoring and molecular characterization of the dengue fever epidemic in Xishuangbanna is important for the prevention and control of dengue fever in Asia.

Sporadic cases have occurred annually since the first dengue fever patient appeared in Yunnan in 2005, and a minor epidemic presented in 2008, with 51 people infected. Since then, the epidemic trend of dengue fever in Yunnan province has become a serious situation. The infected cases grew to 1538 cases in 2013 alone. Then, 331 cases were reported in 2014, including 140 locally infected cases and 191 imported cases^13^,^14^,^15^. In total, 2040 cases of dengue fever were reported in Yunnan province from 2009 to 2014, including 1579 local infection cases and 461 imported cases. The proportion of imported DF cases in Yunnan is the highest of all Chinese cities, of which imported cases from Myanmar and Laos represent the majority (79.83%). This indicates that Yunnan has already become the most serious dengue fever epidemic region of the Chinese inland province. Yunnan is facing an increased risk of an imported DENV outbreak due to its being the main economic port of entry for the countries of Southeast Asia^16^.

Since the beginning of the dengue fever outbreak in Xishuangbanna, Yunnan province in 2015, it is concerning that 10 imported cases appeared, with more than 1000 people infected, yet government statistics about DF infected cases have thus far not been announced. In our study, up to mid-November, a total of 1067 serum samples have been collected from febrile patients, and 852 cases were confirmed to be dengue NS1 positive by the most authoritative local hospital on DENV treatment. Based on previous experience, the viremic period has usually passed for DF patients who have been febrile for more than 5 days, and the success rate of virus isolation and RNA extraction from the serum of these patients is extremely low. In this study, 290 serum samples with febrile course shorter than 5 days were collected to extract DENV RNA, followed by RT-PCR and sequencing, and we had success with 61 of these samples. Meanwhile, 240 samples were selected randomly from DENV patients whose fever courses were between 6–8 days, and only 15 viral RNAs were successfully extracted from the sera at concentrations sufficient for PCR amplification. This is lower than in our past experience, indicating that although the fever course was shorter than 5 days, approximately 79% of the first 290 serum samples had relatively low viral concentration, and this decreased to 6.25% in those with fever course of 6–8 days.

The phylogenetic analysis was conducted primarily based on the envelope (E) protein genes, as most valuable reference sequences from epidemics in Southeast Asia, especially in Laos in recent years, were uploaded to Genbank with only the E gene sequences. In addition, the most important structural protein of the dengue virus is considered to be the envelope glycoprotein, which constitutes the protrusion on the surface of viral particles, organizes the ecotropic virus, mediates the binding of the virus to cell receptors, and induces the generation of specific neutralizing antibodies^17^. Our results show that the epidemic strains of XNDV were evolutionary close to the strains epidemic in India in 2001 and in Sri Lanka in 2004. In addition, the epidemic strains in Fujian,China in 1990 and 2010 were also genetically close to the XNDV strains, which suggests that dengue fever is becoming a locally circulating disease in China.

Although the use of the E gene appears in some studies to analyze DF molecular and evolutionary characteristics in China, there is no assessment of whether DENV E gene is sufficient for molecular epidemiology analysis. As the XNDV epidemic strains has already been proved to be genetically far from DENV of Laos, in the formal analysis, we picked out the reference strains which only contain E gene sequences, such as strains from Laos. The formal phylogenetic analysis was then conducted based on the whole structural protein of DENV, meanwhile more reference strains from China and other Asia countries were involved in the following analysis, as well as the four standard serotype strains of DENV. The tree distribution graph also indicated that the possible origin of the Xishuangbanna epidemic DENV strain were the strains epidemic in India in 2001 and in Sri Lanka, in 2004. Moreover, the results of phylogenetic analysis between the E gene and the whole structural gene appeared the same, which could also be strong evidence in evaluating whether individual dengue E genetic regions can correctly reflect the epidemiological characteristics of DENV in China.

In our study, the transmission relationships between DENV strains in common in Xishuangbanna in 2015 and other regions in China and neighboring Southeast Asian countries were identified, which gives a comprehensive profile of the features of infected populations, the characteristics of epidemic viral strains, and the viral origins including original time or place of Xishuangbanna DF outbreak. Although Xishuangbanna suffered with the most serious DF outbreak in 2015, there are still some infected cases reported in other area of Yunnan. The serotype identification result shows that the epidemic strains in Xishuangbanna belong to the cosmopolitan genotype of DENV-2. This result is different than another study on the epidemiological investigation of DENV in Gengma county in Yunnan province. These two places are geographically close, but the epidemic strains of DENV in Gengma were classified as DENV-1^18^. In that study, 21 local cases and 10 cases imported from Myanmar were positive for DENV-1 through RT-PCR, and 13 strains were sequenced for E genes. Although it is unknown whether other serotypes of DENV epidemic strains exist in Xishuangbanna, all 76 sequences of structural protein coding genes obtained from the epidemic strains in this study were identified as most similar to the serotype 2 standard strain. More research is needed to describe the full picture of the DF epidemic in Yunnan Province in 2015.

Four dengue patients from Burma and one from Laos were treated and cured at the Xishuangbanna Dai Autonomous Prefecture People’s Hospital during the early portion of the DENV outbreak, but RNA extraction failed due to all patients having had a fever for more than 9 days at time of admission. Therefore, the possibility that this outbreak originated from imported patients cannot be ruled out^19^.

There were extensive outbreaks of dengue fever in many areas such as Guangdong, Taiwan, Burma, Vietnam, Laos and other areas in 2015, with different serotypes and genotype strains affecting different areas. Xishuangbanna is geographically near these areas; therefore, the origin of the pandemic strain is relatively complex, warranting a characteristic study on the molecular level. Our study should serve as a reference to follow-up studies focusing on outbreaks of dengue fever in other parts of China and Southeast Asia in 2015.

## Materials and Methods

### Dengue virus identification and RNA Extraction

Serum samples were separated from collected blood, followed by viral RNA extraction. Detection of DENV NS1 antigen was conducted using a rapid one-step dengue fever NS1 test Kit (Blue Cross, Beijing, China). Viral RNA was extracted from 140 μl of dengue virus-infected serum using the QIAamp viral RNA mini kit (Qiagen, Hilden, Germany) and eluted in 50 μl of nuclease-free water.

### Sequencing of structural proteins

Two synthetic oligo nucleotide primer pairs were selected online from Primer-BLAST in NCBI web (http://www.ncbi.nlm.nih.gov/tools/primer-blast/) to amplify the overlapping fragments between base sites 70–2647 of the DENV-2 genome based on the standard strain DEN2SS (GenBank ID: M29095) to obtain the 2325 bp gene sequences of the DENV structural proteins C/prM/E^20^. The primer sequences were as follows: 1-Forward, 5′-TGCTGAAACGCGAGAGAAACC-3′ and 1-Reverse, 5′-CATTGAAGTCGAGGCCCGTT-3′; and 2- Forward, 5′-TGCAGTCGGAAATGACACAGG-3′ and 2-Reverse, 5′-GCCTGCATGATTCCTTTGATGT-3′. All primers were synthesized and purified by an outside vendor (Sangon Biotech Co., Ltd. Shanghai, China).

The One-step PrimeScript^TM^ RT-PCR kit (TaKaRa Co., Ltd. Dalian, China) was used to amplify two overlapping fragments in the virus gene by RT-PCR with the following protocol: initial reverse transcription at 50 °C for 30 min; 35 cycles of denaturation at 94 °C for 30 s, annealing at 55 °C for 30 s, elongation at 72 °C for 1 min and a final elongation step at 72 °C for 7 min. Then, the PCR products were purified and sequenced by Sangon Biotech after identification with agarose gel electrophoresis (AGE).

### Molecular characterization

The gene sequences of structural proteins of the 2015 Yunnan DENV epidemic strains were assembled using Seq Man in DNASTAR version 7.0. The sequences were deposited in the NCBI GenBank database (http://www.ncbi.nim.nih.gov/GenBank/index.html): KX577640-KX577715. Then, the nucleotide sequence substitutions and translated amino acid sequence mutations were analyzed with BioEdit. Predict Protein server (https://www.predictprotein.org/) was used to predict the differences in the secondary structure between the structural proteins of the DEN2SS and 2015 Xishuangbanna epidemic strain^21^,^22^,^23^,^24^,^25^. The amino acid composition and potential protein binding sites were analyzed, and hydrophilicity/hydrophobicity was predicted, as the basis for predicting the exposed and buried regions in the structural proteins. The potential helical structure was also evaluated.

### Phylogenetic analysis and evolutionary analysis

Sequences of the structural protein genes (2325 bp) were aligned using the ClustalX program and compared with the reference strains collected from GenBank under the following accession numbers^26^: Standard strain (DENV-1SS:DQ672562; DENV-2SS:M29095; DENV-3SS:M93130; DENV-4SS:AF326573); China (AF359579; AF276619; FJ196854; KC964094; FJ196852; KC964093; EF051521; KR920365; FJ196853; KC131142; KP723479; KP012546; KP723478; KT187557; KT187554; KT187555; KT187556; KT187558; KT187553; KC964095; FJ196851; AF204177; AF204178; AF119661); Vietnam (FJ461311; JF730049; EU482777; EU482668; EU482788; AB479042); Thailand (FJ898452; GU131886; DQ181799; GU289914; DQ181801); Guinea (EF105378); Sri Lanka (GQ252677); Singapore (EU081178); India (DQ448236); and Colombia (AY702040). Then phylogenetic analysis was performed using Molecular Evolutionary Genetics Analysis (MEGA) software, version 6.0 (Maximum Likelyhood phylogeny test). The nodal reliability of the trees was assessed with bootstrapping (BS) using 500 pseudo replicates^27^,^28^,^29^. Subtree-pruning-regrafting (SPR) was used as the ML search method. Subsequently, Bayesian skyline plot (BSP) analysis in BEAST software was used to build a evolutionary tree, in order to identify the result of the ML tree. To estimate the selection pressure acting on the structural protein gene sequences of DENV, codon-specific, non-synonymous and synonymous substitutions were inferred using the site-model method with PAML4.0^30^,^31^,^32^,^33^.

### Ethical statement

All participants were informed of the study aims, and written informed consent was received from each patient before sample collection. The study protocol was approved by the Institutional Ethics Committee (Institute of Medical Biology, Chinese Academy of Medical Sciences, and Peking Union Medical College) and was in accordance with the Declaration of Helsinki for Human Research of 1974 (last modified in 2000).

## Additional Information

**How to cite this article**: Zhao, Y. *et al.* Molecular Characterization and Viral Origin of the 2015 Dengue Outbreak in Xishuangbanna, Yunnan, China. *Sci. Rep.* **6**, 34444; doi: 10.1038/srep34444 (2016).

## Supplementary Material

Supplementary Information

## Figures and Tables

**Table 1:** Site-specific tests for positive selection of DENV structural protein genes.

Models	lnL	Estimates of parameters	2△l	Positively selected sites
M1	−5207.03	p: 0.89007 0.10993	1.09	NA
		w: 0.00000 1.00000	P > 0.05	
M2	−5208.12	p: 0.89292 0.01928 0.08781		129I
		w: 0.00003 1.00000 1.00000	0.2	
M7	−5189.58	p = 0.00824, q = 0.16024		NA
M8	−5189.48	p0 = 0.99542, p = 0.00800, q = 0.15742	P > 0.05	52Q
		(pl = 0.00458)		129I

lnL, the log-likelihood difference between the two models; 2Δl, twice the log-likelihood difference between the two models; the positively selected sites were identified with posterior probability ≥0.9 using Bayes empirical Bayes (BEB) approach. NA means not allowed.
