# Supplementary material for: Molecular Characterization and Viral Origin of the 2015 Dengue Outbreak in Xishuangbanna, Yunnan, China
Source: Sci Rep. 2016 Sep 29;6:34444. doi: 10.1038/srep34444 (PMC5041078; doi:10.1038/srep34444)
Supplement: Supplementary Information [file srep34444-s1.pdf]

TITLE PAGE

Molecular Characterization and Viral Origin of the 2015 Dengue Outbreak in Xishuangbanna, Yunnan, China

Yujiao Zhao[1,2], Lihua Li[3], Dehong, Ma[3], Jia Luo[1,2,4], Zhiqiang Ma[3], Xiaodan Wang[1,2], Yue Pan[1,2], Junying Chen[1,2], Juemin Xi[1,2], Jiajia Yang[1,2], Lijuan Qiu[1,2], Chunhai Bai[3], Liming Jiang[1,2], Xiyun, Shan[3]*, Qiangming Sun [1,2]*

[1]*Institute of Medical Biology, Chinese Academy of Medical Sciences, and Peking Union Medical College, Kunming 650118, PR China*

[2]*Yunnan Key Laboratory of Vaccine Research & Development on Severe Infectious Diseases, Kunming 650118, PR China*

[3]*Xishuangbanna Dai Autonomous Prefecture People's Hospital, Xishuangbanna 666100, PR China*

[4]*Kunming Medical University, Kunming 650500, PR China*

***Corresponding author:** Qiangming Sun, **E-mail**: qsun@imbcams.com.cn or Xiyun, Shan, E-mail: sgtrhh@126.com

Current postal address: Institute of Medical Biology, Chinese Academy of Medical Sciences & Peking Union Medical College (CAMS & PUMC), 935 Jiao Ling Road, Kunming, Yunnan Province 650118, P.R. China

Telephone number: 86-871-68335165; **Fax Number:** 86-871-68334483

Supplementary Figure

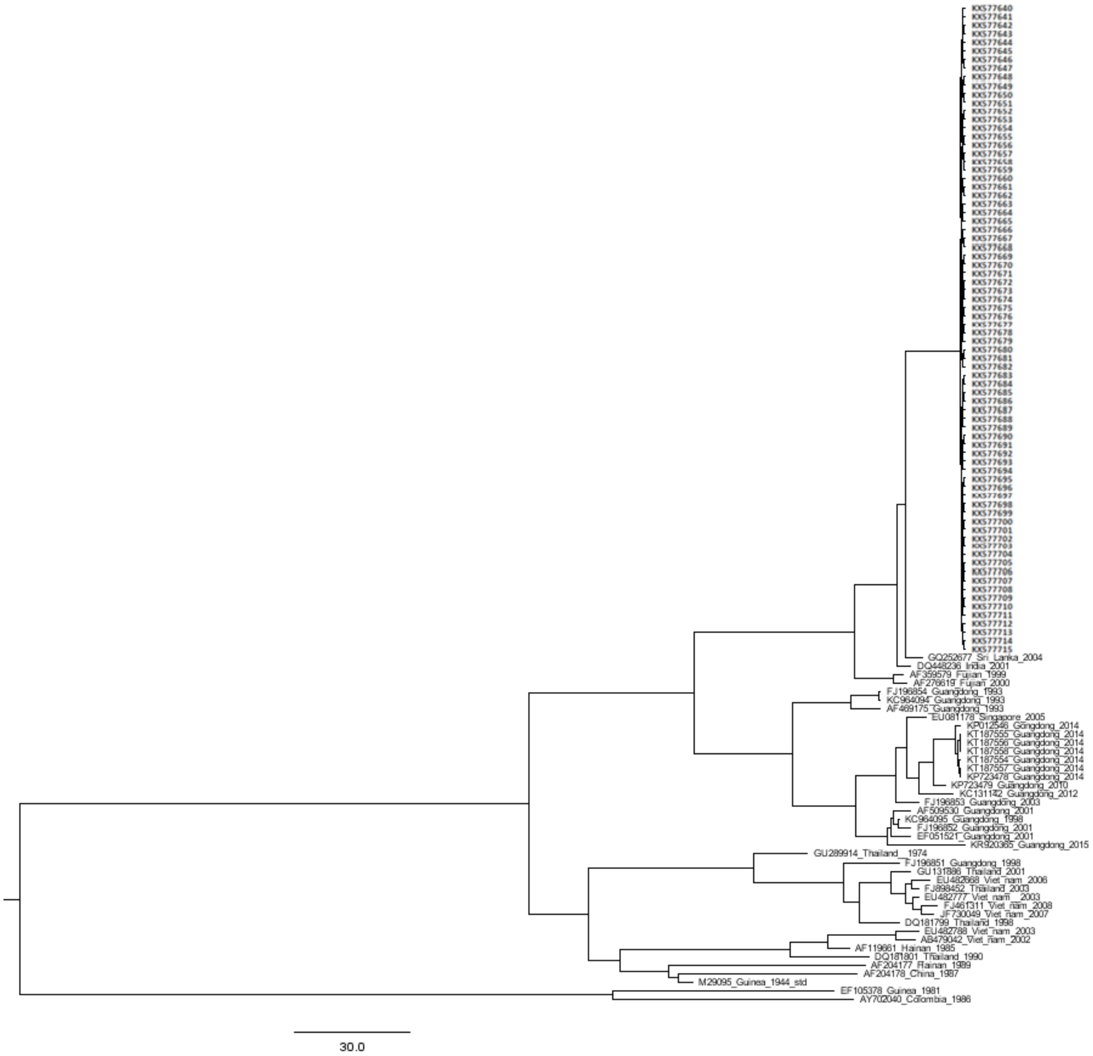

Supplementary Figure 1 Phylogenetic tree of DENV-2 epidemic strains in Xishuangbanna, Yunnan, China 2015. The phylogenetic tree was constructed using the Bayesian skyline plot (BSP) in BEAST software.
